# 

**Published:** 1870-08

**Authors:** 


### CONTENTS.

ORIGINAL COMMUNICATIONS.

Treatment of Exposed Pulps, ........ 329
On Elevating the Dental Profession........ 335
The Employment of Dentists in the Army and Navy ........ 341
Report on Operative Dentistry,........ 348
Filling Teeth with Gold,........ 354
Death of a Pulp from Abscess of an Adjoining Tooth,........ 357

PROCEEDINGS OF SOCIETIES.

Twelfth Annual Meeting of the Indiana State Dental Association,........ 360
Wisconsin State Dental Society,........ 368
American Dental Convention........ 369

EDITORIAL.

American Dental Association,........ 371

